# “A very big challenge”: a qualitative study to explore the early barriers and enablers to implementing a national genomic medicine service in England

**DOI:** 10.3389/fgene.2023.1282034

**Published:** 2024-01-04

**Authors:** Bettina Friedrich, Cecilia Vindrola-Padros, Anneke M. Lucassen, Chris Patch, Angus Clarke, Monica Lakhanpaul, Celine Lewis

**Affiliations:** ^1^ Population, Policy and Practice, UCL Great Ormond Street Institute of Child Health, London, United Kingdom; ^2^ Department of Targeted Intervention and Rapid Research Evaluation and Appraisal Lab (RREAL), University College London, London, United Kingdom; ^3^ Clinical Ethics and Law, Faculty of Medicine, University of Southampton, Southampton, United Kingdom; ^4^ Centre for Personalised Medicine, The Wellcome Centre for Human Genetics, Oxford, United Kingdom; ^5^ Engagement and Society, Wellcome Connecting Science Wellcome Genome Campus, Hinxton, United Kingdom; ^6^ Division of Cancer and Genetics, Cardiff University School of Medicine, Cardiff, United Kingdom; ^7^ London North Genomic Laboratory Hub, London, United Kingdom

**Keywords:** genomic medicine service, whole genome sequencing, implementation research, barriers and enablers, qualitative, service evaluation

## Abstract

**Background:** The Genomic Medicine Service (GMS) was launched in 2018 in England to create a step-change in the use of genomics in the NHS, including offering whole genome sequencing (WGS) as part of routine care. In this qualitative study on pediatric rare disease diagnosis, we used an implementation science framework to identify enablers and barriers which have influenced rollout.

**Methods:** Semi-structured interviews were conducted with seven participants tasked with designing the GMS and 14 tasked with leading the implementation across the seven Genomic Medicine Service Alliances (GMSAs) and/or Genomic Laboratory Hubs (GLHs) between October 2021 and February 2022.

**Results:** Overall, those involved in delivering the service strongly support its aims and ambitions. Challenges include: 1) concerns around the lack of trained and available workforce (clinicians and scientists) to seek consent from patients, interpret findings and communicate results; 2) the lack of a digital, coordinated infrastructure in place to support and standardize delivery with knock-on effects including onerous administrative aspects required to consent patients and order WGS tests; 3) that the “mainstreaming agenda”, whilst considered important, encountered reluctance to become engaged from those who did not see it as a priority or viewed it as being politically rather than clinically driven; 4) the timelines and targets set for the GMS were perceived by some as too ambitious. Interviewees discussed local adaptations and strategies employed to address the various challenges they had encountered, including 1) capacity-building, 2) employing genomic associates and other support staff to support the consent and test ordering process, 3) having “genomic champions” embedded in mainstream services to impart knowledge and best practice, 4) enhancing collaboration between genetic and mainstream specialties, 5) building evaluation into the service and 6) co-creating services with patients and the public.

**Conclusion:** Our findings highlight the challenges of implementing system-wide change within a complex healthcare system. Local as well as national solutions can undoubtedly address many of these barriers over time.

## 1 Introduction

Genomic medicine is transforming healthcare through improved diagnosis (Smedley et al., 2021), improved drug safety and efficacy (Roden et al., 2019), and the prediction of risk (Kamps et al., 2017). In 2018, the National Health Service (NHS) genomic medicine service (GMS) was launched in England to create a step change in the use of genomics in the NHS, including offering whole genome sequencing (WGS) as part of routine care for both rare disease and cancer (NHS England, 2022b). The service capitalizes on the learning and infrastructure developed through the 100,000 Genomes Project, a world-leading initiative set up in England in 2015 with the explicit aim of embedding genomic medicine into clinical care (Genomics England, 2017). For rare disease, WGS has been shown to have improved diagnostic yield over traditional testing approaches such as single gene tests and microarray (Wright et al., 2018). WGS also has a slightly higher diagnostic yield than exome sequencing as it is able to look at etiologic noncoding, structural, and mitochondrial genome variants as well as coding variants which are poorly covered by exome sequencing (Smedley et al., 2021). For cancer, WGS can provide information on etiology, prognosis, and potential therapeutic responsiveness in a single all-encompassing test compared to having to conduct multiple standalone tests arguably making it more cost effective (Turnbull et al., 2018).

### 1.1 Structure of the genomic medicine service

**FIGURE 1:**
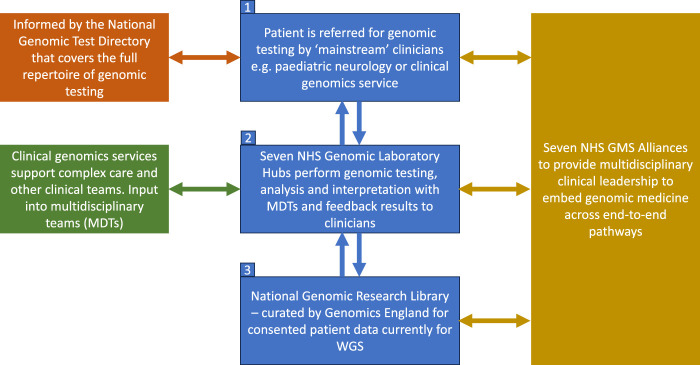
Structure of the NHS genomic medicine service. Adapted from NHS England, 2022b. Contains public sector information licensed under the Open Government Licence v3.0.

The new nationally commissioned GMS, which launched in 2018, is built around seven Genomic Laboratory Hubs (GLHs) and delivered via seven regional Genomic Medicine Service Alliances (GMSAs) whose role is to oversee and co-ordinate the embedding of genomics into routine healthcare across England (NHS England, 2022b) (Figure 1). It is overseen and supported by a Genomics Clinical Reference Group which is tasked with advising on clinical policy and strategy for genomics as well as review and develop guidance and service specifications (including the clinical genetics service specification). As part of the service, WGS is now available as a first-line test for a number of rare and undiagnosed conditions as well as cancer indications (GOV.UK, 2023), including for both pediatric and adult cases (in some cases both), with Illumina Laboratory Services contracted as the WGS provider (Genomics England, 2020). The full repertoire of testing technologies, from single gene testing to WGS are specified in the new National Genetic Test Directory and can be ordered by medically qualified individuals specialized in a sub-discipline other than genetics in both primary and secondary care, thus “mainstreaming” genomics. This is reviewed on an annual basis to ensure it reflects the latest advances in genomics. For WGS, trio testing is recommended for all pediatric referrals and, whilst the whole genome is being sequenced, data analysis is in most cases restricted to a subset of genes relevant to the patient’s features using a virtual panel. Every patient/family undergoing WGS is also offered the opportunity to participate in research and have their data stored in the National Genomic Research Library.

Figure 1 shows the structure of the NHS Genomic Medicine Service (NHS, England, 2022b). The four priority areas, as outlined in the recent NHS England strategy for accelerating genomic medicine in the NHS, is that by 2025, genomic medicine will be 1) fully embedded across multiple clinical pathways in the NHS, 2) will deliver equitable genomic testing to improved prediction, prevention, diagnosis and precision medicine, 3) will enable genomics to be at the forefront of the data and digital revolution, and 4) will incorporate the latest cutting-edge science, research and innovation (NHS England, 2022b). To do this, the NHS is embedding the patient voice across the GMS infrastructure and governance, and there is patient representation in each of the seven GMSAs, the Genomics clinical reference group as well as on the Genomics Programme Board (NHS England, 2022a). Another key focus is around developing the workforce and each GLH is funded to include a multidisciplinary clinical and scientific leadership infrastructure led by a Medical, Scientific, Operations and Informatics Director. Similarly each GMSA is led by a Clinical Director with input from a range of professionals including clinical leads for different areas such as cancer, nursing and midwifery, as well as a dedicated Research Director (NHS England, 2022a). Each GMSA has also been funded to deliver a range of regional transformational projects including embedding implementation of familial hypercholesterolemia (FH) services in primary care and delivery of a comprehensive service for the detection of Lynch syndrome (NHS England, 2022a).

### 1.2 Integration of genomics into healthcare

Effective integration of genomic medicine into a complex healthcare system such as the NHS requires system- and organizational-wide change. These include, amongst other aspects, the development of healthcare and electronic systems to manage data and workflows, technical and bioinformatics infrastructure to process, sequence and analyze samples, and sufficient workforce capacity and capability to counsel, request, interpret and communicate results to patients and families (Pearce et al., 2019; Raspa et al., 2021; Walton et al., 2022). Mainstreaming of genomics will also require upskilling of non-genetic specialists so that they too can counsel, request and communicate genomic results to patients (Barwell et al., 2019), as well as cross-discipline discussions-for example, through multidisciplinary team (MDT) clinics (Best et al., 2021b) and MDT case discussion meetings.

**FIGURE 2:**
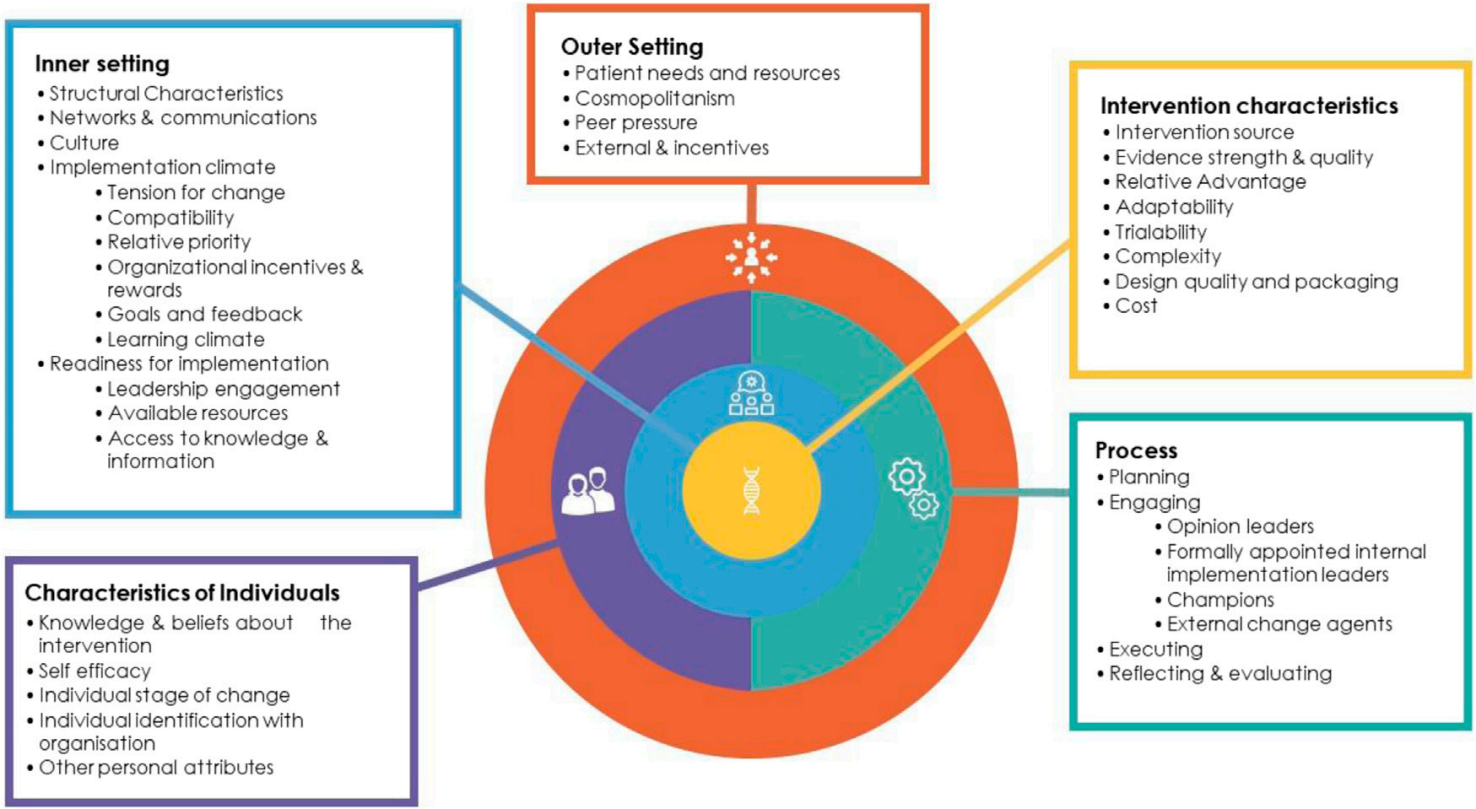
Consolidated framework for implementation research (CFIR) (Damschroder et al., 2009). List of the five domains (Intervention characteristics, Inner setting, Outer setting, Intervention characteristics, Characteristics of individuals, Process) and underlying constructs. Reproduced from Best et al. (2021a), licensed CC BY 4.0. No changes were made to this figure. A copy of the license can be found at http://creativecommons.org/licenses/by/4.0/.

Across the globe, national genomic-medicine initiatives are underway; however different approaches are being utilized and local healthcare contexts vary (Pearce et al., 2019; Stark et al., 2019). For example, In Australia, genomic medicine is being implemented in an incremental manner (unlike in the UK), with policy decisions on clinical test funding occurring variously at the healthcare service, state and national level. In order to ensure implementation of genomic medicine is effective, it is important to understand the organizational, social and cultural factors involved across different countries, and to share strategies and experiences, including both barriers and enablers. Implementation science frameworks promote a theory-driven and systematic approach to evaluate the implementation of interventions in real-world settings (Bauer et al., 2015). The Consolidated Framework for Implementation Research (CFIR) (Damschroder et al., 2009) is a comprehensive framework comprising evidence-based factors that act as barriers and enablers to the successful implementation and adoption of complex programs (Damschroder et al., 2009). The framework provides a taxonomy of 37 operationally defined constructs organized into five major domains: 1) Intervention Characteristics (e.g., evidence strength and quality); 2) Inner Setting (e.g., culture); 3) Outer Setting (e.g., external policies and incentives; 4) Characteristics of Individuals (e.g., knowledge and beliefs about the intervention); and 5) Process (e.g., executing) (see Figure 2). We used the CFIR to identify organizational and system-level factors that are impacting the implementation of the GMS in England. We asked the question, “what are the barriers and enablers to implementing the GMS for pediatric rare disease diagnosis, during the early years of the service?”.

## 2 Materials and methods

Our study was situated within a pragmatist paradigm, meaning that we sought functional knowledge to produce positive change (Weaver, 2018). NHS ethical approval for the study was granted by London-Bloomsbury Research Ethics Committee (21/PR/0678).

### 2.1 Sample and recruitment

Qualitative semi-structured interviews were conducted with key stakeholders across England who are involved in the implementation of the Genomic Medicine Service (GMS). These included those involved in designing the service—known from hereon in as “designers”, and those tasked with delivering implementation across the GMSAs and GLHs–known from hereon as “implementers”. We aimed to interview two implementers from each of the seven GMSAs and/or GLHs and used purposeful sampling to ensure participants covered a range of professional roles. Potential “designers” were identified through the project advisory team, comprising representatives from policy, practice, and patient organisations. For the “implementer” interviews, the medical or clinical director of each GMSA and GLH was approached and invited to take part in an interview or nominate another member of the leadership team. Potential participants were approached via email (CL) with an information sheet explaining the purpose and procedure for the study.

### 2.2 Data collection

We used the CIFR to inform the development of our interview schedule (Appendix 1) and explored the organizational and system-level barriers and enablers in implementation. As we were focusing on the organizational and system levels, we did not include questions that focused on Characteristics of Individuals (Domain 4), but rather we focused on Intervention Characteristics (Domain 1), Outer Settings (Domain 2), Inner Settings (Domain 3) and Process (Domain 5). Two slightly different interview schedules were used for the two groups of interviewees.

All interviews were conducted by CL other than one that was conducted by BF, between October 2021 and February 2022 and recorded using online videoconferencing software following participant consent. Interviews lasted between 28 and 64 min (median = 53 min), were fully transcribed, de-identified and uploaded to NVivo 12 (QSR International Pty Ltd, 2018). Participants were given an identifying code, e.g., P1, P2, etc.

### 2.3 Data analysis

We conducted a thematic analysis using a codebook approach (Roberts et al., 2019) containing CFIR constructs. First, interview transcripts were checked against the audio by BF and identifying information was redacted followed by data familiarization. Next, the initial codebook containing CFIR domains and constructs was developed by BF, including tailored definitions of each CFIR construct to fit the context of the GMS. This was followed by independent coding (BF and CL) of the same four transcripts. Findings were discussed and adaptations to the codebook were made, including removal of some codes which did not fit the data or where there was too much cross-over in the context of the GMS (e.g., Cosmopolitanism and Engaging), and at this stage a number of inductive codes were also added, e.g., COVID. It was also decided at this stage to remove the code “Complexity” from the codebook because we found that nearly all the data could be coded here, making it too varied in its content and therefore unhelpful (as a result of implementing a complex service into an existing complex health system). The total dataset was coded by BF using this version of the codebook, which included 17 of the original CFIR constructs and 1 newly established construct (COVID). In the next stage of analysis, the codes were reviewed by CL and BF, and inductively derived subcodes, some of which represented barriers and some of which represented enablers, were then applied by BF to the CFIR constructs, e.g., “Improved equity of access” (enabler) for the Category “Relative Advantage”. These subcodes were then discussed between BF and CL and some minor changes to wording were made. All transcripts were then re-coded in NVivo by BF using this final codebook. In the final stage of analysis, a narrative structure was created for each domain. In some instances, we “clustered” more than one CFIR construct together where the constructs aligned or were inter-related. For example, we clustered “Intervention Source” and “Trialability” together because the idea for the GMS originated as a follow-on from the 100,000 Genomes Project (“Intervention Source”), and at the same time, a key aim of the 100,000 Genomes Project was to pilot the implementation of genomic medicine in the NHS (“Trialability”). It therefore made sense narratively to describe these two constructs together because they were so closely connected. Below we present the main barriers and enablers along with supportive quotes.

## 3 Results

### 3.1 Participation characteristics

One person declined to participate (reason unknown), one did not respond to the email and one agreed, but a convenient time could not be found. In total, 21 people took part in an interview (87% response rate). This comprised 7 designers (including representatives from Genomics England, Health Education England, NHS England and Department of Health) and 14 implementers (including the following named role: Medical Directors, Clinical Directors, Medical Leads, Lead Scientists, Laboratory Leads, Education and Training Leads, Operational Directors), including two from each of the seven GMSAs/GLHs (n = 8 from GLHs, n = 6 from GMSAs). Participant characteristics can be found in Table 1.

**TABLE 1:** Participant characteristics.

Characteristic	N (%)
Gender	
Female	13 (62%)
Male	8 (38%)
Age	
Range	30–60 years
Mean	50 years
Median	51 years
Professional background Implementers	
Clinical genetics consultant	7 (50%)
Clinical/research scientist	7 (50%)
Role - Designers	
Educationalist	2 (29%)
Genetics/Rare Disease advisor	2 (29%)
Policy and strategy	2 (29%)
Informatics specialist	1 (14%)

### 3.2 Barriers and enablers

In total, 17 of the CFIR constructs and 1 new construct (COVID) were identified as barriers and/or enablers to the success of the GMS during the early years of the service. In the vast majority of cases, CFIR constructs were identified as both barriers and enablers, highlighting the interconnectedness of the various approaches impacting on the implementation of a complex service (the GMS) within a complex system (the NHS).

#### 3.2.1 Intervention characteristics—attributes of the GMS which influence the success of implementation

##### 3.2.1.1 Intervention source—where the idea for the GMS originated; trialability—piloting of service prior to implementation

These two constructs were closely aligned in that they related to participants’ descriptions of the 100,000 Genomes Project. Participants acknowledged the value of the 100,000 Genomes Project in acting as a pilot for bringing WGS into the NHS and for providing the foundations and evidence-base which enabled clinicians and scientists to “work through a lot of the 100,000 Genomes Project complexities together” (P9, designer). They also acknowledged the developments that had gone on within genetics more broadly as providing the backdrop to the GMS—“its been an evolution over many years” (P5, designer). Nevertheless, despite the 100,000 Genomes Project, participants reflected on the lack of opportunity to pilot the new GMS before rollout, meaning that services were “learning as we go” and staff were having to be “a little bit more reactive to what is happening rather than being able to be proactive” (P1, designer).

The volume of WGS tests was perceived by some to have been scaled up too quickly resulting in concerns about the capability and capacity of the service to cope due to lack of sufficiently trained staff and volume of work. In particular, some queried whether the service needed a period of “stabilization” before implementing any further change, including time to evaluate the benefits of WGS.

“I think the 100,000 genome project was great, it was exciting, created lots of exciting challenges. I’d ask myself the question what did we actually learn from that? What we should have done in that window is say OK let’s use this [100,000 Genomes Project], let’s really assess, properly assess the efficacy in certain conditions for whole genomes.” (P11, implementer)

##### 3.2.1.2 Relative advantage—perceived advantage of the GMS over what was available previously; evidence strength and quality—validity of evidence supporting the GMS; cost implications—cost associated with genomic testing/WGS

These three constructs were inter-related in that the validity of the evidence supporting the use of genomic medicine for rare disease diagnosis, which in part came from the 100,000 Genomes Project, underscored the advantages of setting up the NHS GMS and rolling out WGS as a first-line test in certain cases. The potential cost-effectiveness of WGS was also perceived by many as being advantageous to the NHS in the long-term.

Participants identified a number of advantages of the new GMS over previous iterations of the service. These included: less fluctuation in the quality of the service and improved equity of access as a result of standardization meaning that “patients are getting better access to proper comprehensive testing” (P20, implementer); perceived increased diagnostic yield particularly as a result of WGS; improved access to therapies and treatment; and reduced costs overall as a result of “economies of scale” (P16, implementer).

However, in term of barriers, WGS was perceived as having a number of disadvantageous knock-on effects including increased turnaround times with patients “waiting 6 months” for results (P18, implementer), and a reduction in the number of patients being tested because where a clinician could previously order exome sequencing, they were now obliged to order WGS, meaning that some patients weren’t being tested who would have been tested previously because of a reluctance from health professionals to consent and order WGS tests. Some participants queried whether we should be offering WGS as a first line test in certain cases, in particular those cases where the clinical indication was clear, with Noonan syndrome being cited as one example. The rationale behind the decision to offer WGS over exome sequencing was also questioned by a few participants, in particular whether it was politically or scientifically driven, as exemplified in the following quote:

“We don’t have enough evidence to prove that at this stage [WGS] is the right test to replace a number of other tests, both in terms of cost and turnaround times, and clinicians’ effort to access it…I suspect this was mostly stimulated by the need to make some strong political announcement rather than based on strong clinical or scientific evidence.” (P18, implementer)

**Barrier: WGS perceived as being politically rather than clinically driven**.

A common viewpoint was that WGS had been given far too much focus by those designing the service where “90% of the discussion are around whole genome sequencing and that makes up maybe 5% of all the work that goes on” (P10, implementer). The specific consent and test ordering procedure required to offer WGS was perceived to have “created even more layers into a consultation” (P12, implementer), and could potentially include “seven forms for one trio test, which a lot of people find very difficult” (P21, implementer). One participant described there being a lot of “to-ing and fro-ing” (P16, implementer) between laboratory staff and clinicians whilst waiting on bloods (as trio testing requires samples from both the patient and both parents where possible) or as a consequence of having to return incomplete forms “resulting in a backlog of patients” (P16, implementer).

**Barrier: onerous administrative aspects required to consent patients and order WGS tests**.

In order to cope with the additional workload required around WGS, a number of sites had employed “genomic associates” who often had not had any formal genetics training, to support the consenting process (including discussion around the diagnostic testing and research participation), freeing up clinicians to focus on clinical appointments.

**Enabler: employing genomic associates to support the consent process**.

##### 3.2.1.3 Design quality and packaging—how the GMS is built and delivered

Consolidation of services into the seven GLHs and GMSAs was generally regarded as a positive change that facilitated the standardization of high-quality services across the country and changed relations with laboratories so that they were “now hav [ing] to work as a network” (P15, implementer). Moreover, the governance structures and leadership teams that have been put in place were perceived as being effective in bringing people together, as well as ensuring that guidelines and operational procedures “trickle down to all the different groups” (P1, designer). Nevertheless, a number of negative outcomes of consolidation were acknowledged, mainly in relation to their set up which was described by some as having been a “torturous” (P13, implementer), and “painful” (P15, implementer) experience, and had created “disruption” (P17, implementer) with some laboratories closing, some people losing jobs, and had created unnecessary competition with services pitching against each other:

“With genomics there’s always been a really excellent collaborative way of working and I think that has been diminished to some degree over the last few years by who’s recruited the most patients [which] doesn’t necessarily create or foster an environment where you’re looking to support each other.” (P10, implementer)

The new National Genomic Test Directory (NGTD) was another key feature of the GMS viewed as a positive advancement overall. The R-numbers—the codes used to order a clinical test for a rare disease - had been adopted quickly and were perceived as a “good currency…that’s enabled the scientists to work better across the laboratories” (P4, implementer). However, some commented on the poor functionality of the directory, e.g., “It’s quite clunky” (P11, implementer), and that there was acknowledged to be a disconnect between adding new tests to the directory and the laboratory being able to deliver them. With regards to the mainstream agenda, notable perceived benefits were that it would free up clinical geneticists to deal with more complex cases and “speed up the patient journey” (P17, implementer). Nevertheless, getting to that stage was identified as “a very big challenge” with some clinicians having “really embraced genomics” (P19, implementer), whilst others were “genuinely concerned because it’s not something they routinely do” (P11, implementer). One participant queried whether the pace with which the mainstreaming agenda was being pushed was too fast: “We might realize that we were a bit too brave with that” (P18, implementer).

**Barrier: mainstreaming agenda encountered reluctance from those who did not see it’s a priority**.

The National Genomic Information System (NGIS), the online test ordering tool for WGS, received widespread criticism from participants. Envisioned as a standardized digital system - “the holy grail for the service to work well” (P11, implementer) - it was in fact “very paper based” resulting in the need for administrative support.

“We’ve had to employ a lot more admin people and genetic technologists in essence to handle sample receipt and communication with clinicians, and input into NGIS” (P16, implementer).

**Enabler: employing support staff to support the test ordering process**.

Whilst a digital infrastructure was perceived as a priority, the reality was that NGIS didn’t integrate with local IT systems, raising concerns that this would lead to divergence amongst laboratories with each one “looking for its own individual solutions” (P9, designer).

**Barrier: the lack of a digital, coordinated infrastructure to support and standardize delivery**.

The National Genomic Research Library was perceived as an important aspect of the GMS in order to “drive improvements in genomics going forwards” (P5, designer), and to enable patients with rare diseases to participate in research. Nevertheless, facilitating patient consent to take part was identified by some interviewees as tricky, in particular because consenting for diagnostics and for research was expected to happen during the same appointment, with one participant commenting that “a lot of people haven’t bothered to do that.” (P14, implementer).

#### 3.2.2 Outer settings II—the setting within which the inner setting exists

##### 3.2.2.1 External policies and incentives—national factors that determine and guide the roll out of the GMS

England was perceived as well placed to deliver the GMS, and in particular WGS, because of four notable key factors. These included 1) the political will driving the GMS, 2) world-leading technology available within England to sequence whole genomes, 3) professional expertise, and 4) coordination:

“If there is a place where whole genome sequencing can be implemented clinically without encountering big risks, this is probably the UK because of the coordinated approach, the general level of expertise.” (P18, implementer)

Yet the reactive way in which the rollout had occurred caused concern amongst a number of interviewees, with one participant commenting that “a lot of the assumptions that were baked into the original tender actually failed to materialize” which meant services had “to replan and reorganize and redeliver” (P6, designer). There was also notable dissatisfaction amongst some interviewees relating to the relationship between those dictating policy and those on the ground delivering the service, which some perceived as being too “top-down”:

“The seven GLHs are told what to do whereas before we weren’t. So we’d sit in a room and make mutual decisions…now we sit in a room and we’re told what to do…for good or bad”. (P15, implementer)

Top-down pressures and overambitious targets around WGS were described as having “made life really quite difficult for us by, in a sense, turning on the pressure to deliver very early on, which we’ve not been able to do for a whole variety of complex reasons.” (P14, implementer). Participants reflected that whilst policymakers had signed a large contract with the biotechnology company Illumina to sequence hundreds of thousands of genomes over 5 years, the reality on the ground was that there currently was not the capacity—“we just don’t have the people power” (P12, implementer) - nor investment from the NHS to deliver on this target in a timely way, meaning that patients were waiting longer than anticipated to receive their results: “the turnaround times for whole genomes are slipping massively” (P19, implementer).

**Barrier: the timelines and targets set for the GMS were perceived by some as being too ambitious**.

##### 3.2.2.2 Cosmopolitanism—the collaboration between different groups within the NHS in England to facilitate the roll out

Examples were cited of interactions taking place at both a local and regional level. This included “lots of working groups [which have] sprung up where there weren’t any before” including a “Rare Disease Working Group that meets once a month” (P6, implementer) to progress research. It was acknowledged that collaboration across GLHs/GMSAs facilitated “a very much joined up approach to make sure that we’re all working towards this common goal of having the GMS work” (P1, designer). Those implementing the service from an operational capacity also reflected on how the networking between sites had facilitated “good working relationships” (P11, implementer).

##### 3.2.2.3 Patient needs and resources—what the service should provide for patients and how patients can be involved in the roll-out process

A number of examples of patients being proactively involved in implementation activities and decision-making were cited. These included patient panels contributing to business plans including GMSAs’ equality, diversity and inclusion strategies, patients being involved in preparing educational material, and patients advising on the language used in WGS-based diagnostic reports “so that it’s accessible to both patients and clinicians” (P14, implementer). One interviewee acknowledged the importance of having patient input into the design of the service in order to “make sure we’re retaining particularly the patient trust in what we’re doing and how their data is used” (P5, designer).

**Enabler: Co-creating services with patients and the public**.

##### 3.2.2.4 COVID—impact of pandemic on rollout of the service

Unsurprisingly, COVID-19 had a negative impact on the rollout of the GMS, including delays to the rollout of the service. Furthermore, COVID-19 caused “…reagents and consumable supply issues” (P12, implementer). Interestingly, however, COVID had some positive side effects. The pandemic highlighted the benefits of having a national network of laboratories, for example, for pooling resources; it created a need for finding innovative ways of working—for example, digital meetings due to social distancing, which had continued post-pandemic; and it highlighted the resilience of the service which was “able to continue” and “to be able to begin the rollout of whole genome sequencing” (P5, implementer).

#### 3.2.3 Inner settings—characteristics of the implementing organization

##### 3.2.3.1 Culture—NHS norms, values and ability to change and adapt; implementation climate—the collective attitude to change and the willingness to implement genomic medicine

Culture and Implementation Climate were closely intertwined, particularly because the complexities of creating change in an organization such as the NHS directly impacted participants’ attitudes and willingness to be part of that change. Interviewees described tensions between the policy-level vision of genomics and the organizational capability to deliver on that vision. Whilst participants were unanimous in their agreement that genomic medicine could yield significant benefits to patients, embedding genomic medicine within the NHS was perceived as challenging for a number of reasons. First, it is taking place within a “complex ecosystem” where genomics isn’t seen as a priority for many non-genetic specialists, who are understandably preoccupied with dealing with “huge waiting lists” (P17, implementer), have limited time and resources, and are grappling with significant post-pandemic fatigue meaning that they are understandably reluctant, even resistant, to accept change.

“I think perhaps we were slightly overoptimistic that we would be working in an environment that would be receptive and ready to go.” (P14, implementer)

Secondly, it requires adaptations to local processes and practices that have built up over many years, and there is a reluctance from mainstream clinicians to take on genetic testing as part of their role:

“they’ve always referred to clinical genetics for genetic testing and they’re questioning why they can’t do that still” (P17, implementer).

**Barrier: the “mainstreaming agenda” encountered reluctance from those who did not see it as a priority**.

Genomics was perceived as a “hard sell”, in particular because of the additional time required to consent patients for WGS, as highlighted by the following comment:

“We’re immediately going in with a suboptimal sell to the clinicians to say here, when you want to order a test, but don’t order it like you would normally, don’t just tick the box and send it off, we need you to fill in these three forms and do this extra ten-minute discussion,” which is a very hard sell. We need to go in there and convince of the benefit at the same time as apologizing for the inefficient process and say “look, we’re going to try and smooth that up and ease that up as we go forwards”. (P19, implementer)

**Barrier: onerous administrative aspects required to consent patients and order WGS tests**.

Yet despite these concerns, some participants reflected that change on such a significant scale would take time to embed, as articulated through the following quote:

“It’s recognizing that genomics transformation, it will happen more and more over a greater period of time, it’s not something you can just snap your fingers and have it all done within two years, you'll have some big improvements within two years, but actually a lot of it will mature and you're getting greater benefits over time. I think we just need to recognize that and understand that.” (P6, designer)

In particular, a number of interviewees spoke of the need to win “hearts and minds”, i.e., convince clinicians around the value of embedding genomic medicine within their clinical practice. Participants also reflected on examples of where individuals have been receptive to change. There were examples of leadership support from within Trusts, as well as an acknowledgement that, despite some initial hurdles, the GLHs/GMSAs were “now working well together” (P13, implementer). The importance of having “genomic champions” across various mainstream disciplines, to drive this change in culture through raising awareness and supporting the mainstreaming agenda was seen as critical.

**Enabler: having “genomic champions” embedded in mainstream services to impact knowledge and best-practice**.

##### 3.2.3.2 Readiness for implementation—practical preparedness for the changes necessary to implement genomic medicine; structural characteristics—logistic, physical aspects, equipment that are relevant for the roll out

Readiness for Implementation and Structural Characteristics were closely linked because frequently structural aspects such as physical space and technical infrastructure directly impacted whether or not participants felt that they and/or their departments were ready for implementation.

A commonly cited view was the important contribution staff had collectively played in ensuring that the service was ready for rollout, as highlighted by the following quote:

“I think it’s people actually. I think it’s all down to people and I think people have worked really, really hard and … with some complaints of course because it has made our lives hugely more challenging.” (P19, implementer)

From an organizational-level perspective, capacity-building and upskilling the workforce (engagement events, online training, etc.) were key enablers to ensuring the service was ready to deliver on the promises of the GMS. This work incorporated education and training such as online courses, films showcasing the importance of genomics for various clinical specialists, “bitesize pieces of information, videos, podcasts, short courses, introductory courses…” (P2, designer) developed by Health Education England (https://www.hee.nhs.uk/) as well as the work conducted by the GLHs and GMSAs, in particular the Education and Training Leads, to increase awareness of genomics across different medical specialties. Examples of capacity-building through locally developed training routes for clinical scientists, bioinformaticians and technologists were also cited:

“We’ve trained up our own hybrid trainee clinical scientists as preregistration scientists…So they come in with experience and we’ve put a four year training route in place for them so they work at different parts of the lab and then go onto registration that way.” (P16, implementer)

“Hands-on” support, for example, deploying genomic associates for a period of time to train clinical teams in the consenting process, and running educational multi-disciplinary team meetings, were also cited as pro-active ways to support the mainstreaming agenda. Yet these initiatives ran into obstacles including lack of time for clinicians to take part in training, and a perceived lack of confidence amongst non-genetic specialists in ordering genomic testing:

“I think a lot of clinicians can be in some cases a little bit hesitant to use these new technologies just because they don't feel confident in their own knowledge and skills in this area.” (P1, designer)

**Enabler: capacity-building**.

Participants raised concerns about a lack of trained specialists to take on roles within the GMS, particularly naming the lack of clinical scientists and genetic counsellors, and the GMSA/GLH leadership roles were perceived as having taken people away from frontline jobs and thereby adding further pressure to clinical genetics services.

**Barrier: lack of trained and available workforce to seek consent from patients, interpret findings and communicate results**.

Whilst some genetic departments had employed staff (such as genetic counsellors, genomic associates, or additional administrative staff) to deal with the increased workload resulting from WGS, others noted the lack of financial support from hospital management and/or central funding for new roles, which meant that highly qualified staff were instead spending time doing administrative work.

“We very much recognize the need for a group of staff who can chase up samples, chase up consent, chase up paperwork and leave all of that stuff—or take all of that stuff—away from the scientists who should be doing the scientific work.” (P15, implementer)

Moreover, frustrations relating to poor technical infrastructure and interoperability of systems for managing genomic data were described including delays to the delivery of software tools to manage genomes, reliance on paper reports for processing samples, results being returned to clinicians via email, and a lack of standardization of electronic patient records which created challenges in accessing patient information. “Clinicians will be sat at their desks and they want to look at their genetic results, and they’re not able to do that. They have to look through their emails to find a PDF attached to an email, so our genomic results are not going in the patient record and it’s not just a challenge. It’s a clinical risk because they’re not able to find those genomic results.” (P13, implementer).

**Barrier: the lack of a digital, coordinated infrastructure in place to support and standardize delivery**.

In terms of structural characteristics, notable benefits of having centralized laboratory services were that it had resulted in less unnecessary duplication of services across regions and was driving automation in order to deal with the volume of samples. However, the lack of capital investment and physical space to absorb the increased workload was identified as a current barrier:

“No one of our laboratories could stand up and absorb all of that work because there simply isn't the estate to do that.” (P11, implementer).

##### 3.2.3.3 Networks and communication—formal and informal networks and communications within a GLH or GMSA

An important factor driving the perceived success of the GMS was the collection of various networks and communication channels that had been established, some of which had been set up since the formation of the GMS, such as regional MDTs.

“What we’re also developing is regional MDTs. We’ve got one already that’s been running for ages for our cardiac and respiratory tests and we’re in the process of setting up and developing regional MDTs for dermatology, neurology, adult and paeds etc.” (P21, implementer)

Some interviewees described how there was now “more of an interaction with other disciplines, other specialties” (P12, implementer) largely driven by the mainstreaming agenda. Participants also reflected on the communication that had happened with the smaller, peripheral laboratories, where conversations had been “very open” with how work would be apportioned.

**Enabler: enhancing collaboration between genetic and mainstream specialties**.

#### 3.2.4 Process—activities and strategies used to implement the innovation

##### 3.2.4.1 Engaging with external persons (including for education and training purposes)

Participants described high levels of engagement between senior management and the NHSE Genomics Unit (tasked with providing strategic oversight and performance monitoring) and was considered important to the success of the rollout. This included weekly meetings, “various other meetings, partnership board meetings, etc.” which were described as being “clear” and “keeping us on track” (P12, implementer). There was also an acknowledgement that NHSE were able to provide the administrative support required to support the GMS, for example, the national working groups which had emerged out of the GMS.

“Having the Genomics Unit means that they’re programmed effectively. In the past that would have been a very keen person to set up a group and run it…where the Genomics Unit and NHS England come in and do all of that for us.” (P16, implementer)

Examples of interactions between senior leadership and external organizations who were perceived as being key to embedding genomics across a range of clinical specialties, for example, the Nursing and Midwifery Council and The British heart Foundation, were also highlighted.

Yet the complexities of communicating across the entire GMS network were acknowledged, with a perceived over-reliance “on information trickling down to people…if you don't happen to be in the right email train you might not necessarily get the information that you need.” (P1, designer). Engaging with clinical teams about a “very broad range of operational priorities across an enormous range of different organizations” (P14, implementer) was also identified as challenging. Implementers highlighted the difficulties they faced in both serving their own Trust as well as delivering for national bodies such as NHSE, who sometimes have different requirements and priorities, with one participant describing the situation as feeling like “my mum’s telling me to do one thing, my dad’s telling me to do something else, and I want to please both of them but sometimes I can’t” (P11, implementer).

##### 3.2.4.2 Execution—steps related to accomplishing the roll out as planned

Participants queried whether the plans for the newly commissioned GMS had been unrealistic and overambitious, as highlighted by one interviewee who commented that:

“ I think there was a lack of understanding on the NHSE side about what would have to be in place before we could start offering it diagnostically, there was a great enthusiasm to get it launched and we were being asked to educate the users and tell them it was happening and it just didn’t match up to reality.” (P4, implementer)

Yet it was acknowledged that there was a delicate balancing act being played out between ensuring clinical teams were able to access and offer testing to their patients, and at the same time, the GLHs having the infrastructure as well as validation achieved to be able to deliver those tests. Further issues impacting the execution of the service included delays in recruiting for key management posts and a lack of trained clinical scientists to conduct data interpretation.

**FIGURE 3:**
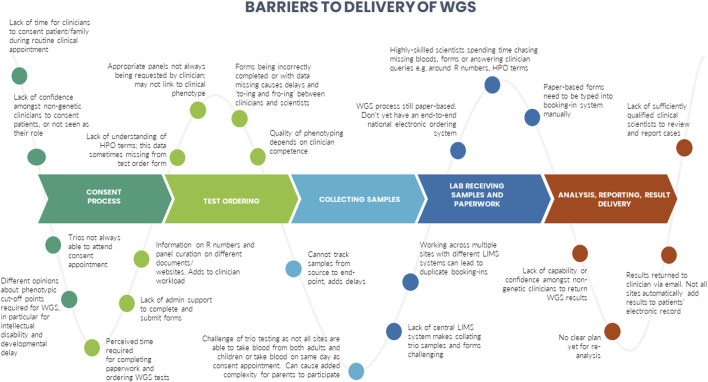
Identified barriers to the delivery of whole genome sequencing.

**FIGURE 4:**
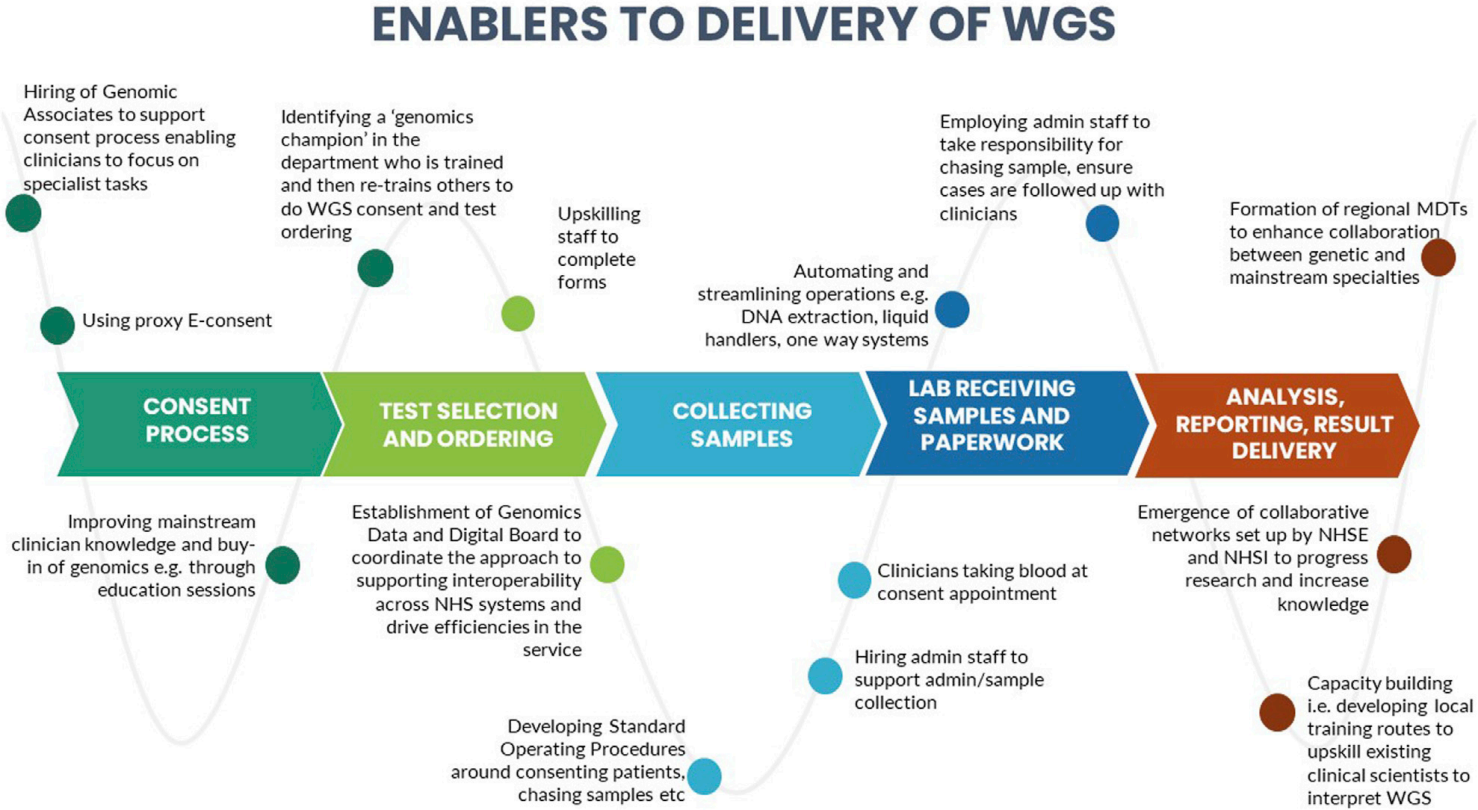
Identified enablers to the delivery of whole genome sequencing.

A number of barriers and enablers related to the delivery of WGS in particular across the testing pathway were identified during the interviews. These are presented in Figures 3, 4.

##### 3.2.4.3 Formal Reflection and Evaluation - implementation quality monitoring, internal feedback

Progress towards the aims and ambitions of the GMS, including equity of access for patients were being monitored in a number of ways both in-house and nationally. Sites were conducting their own audits including postcode mapping as well as ethnicity monitoring in order to enable them to target those areas where they weren’t receiving samples, or those ethnicities under-represented. Patient level contract monitoring, the format through which the activities of individual sites was captured nationally, was also highlighted as an important component of ensuring equity of access, and efforts had gone into ensuring there was a uniform format for collecting data (e.g., turnaround times, geographies, patient characteristics) so that “we can get a much better idea of actually what’s being delivered across the country.” (P5, designer).

**Enabler: building evaluation into the service.**

### 3.3 Differences between designers and implementers

We identified certain CFIR constructs that appeared to be of greater relevance to one group over the other. Designers were more focused on the perceived benefits of the “Design Quality and Packaging”, such as the NGTD, NGIS, and governance and leadership structures. They looked to the future, noting the importance of the longer-term aims of the GMS including capacity building in order to “mainstream” genomics (“Readiness for Implementation”). They discussed the importance of evaluating the service in order to deliver on a key ambition for the service - equity of access (“Formal Reflection and Evaluation”). Many of the statements were therefore what one might describe as “goal orientated”. Implementers, understandably, made more comments that were “process-orientated” because they related to the lived-experience of delivering the GMS. For that reason, frequent areas of discussion related to the Inner Setting and Process domains. Implementers described many of the challenges identified in the Readiness for Implementation and Structural Characteristics constructs, including logistical, turnaround times and workforce challenges. Execution was a frequently coded theme for this group, with participants identifying numerous day-to-day barriers to delivering the GMS in an already overstretched system (as detailed in Figure 3). In comparison, the enablers (detailed in Figure 4) were identified by both Implementers and Designers.

## 4 Discussion

Whilst previous studies have looked at the readiness of the NHS to deliver genomic medicine (Pearce et al., 2019), to our knowledge, this is the first qualitative study conducted in England since the launch of the new GMS in 2018 to examine the barriers and enablers to implementation during the early years of the service. Our findings highlight that those involved in the delivery of the GMS strongly support the aims and ambitions of the service and are clear about the benefits of using genomic technologies for rare disease patients.

Nevertheless, there appears to be a current mismatch between the vision for the GMS (NHS England, 2022b), and the service which is currently being delivered. Through our interviews (particularly those conducted with service “implementers” i.e., those on the ground who may be closer to the day-to-day practicalities of delivering the service), we identified: 1) concerns around the lack of trained and available workforce to deliver the service as intended, both in terms of seeking consent, interpreting findings and communicating results; 2) significant challenges in relation to having a coordinated, digital infrastructure in place to support and standardize the delivery of the service with significant knock-on effects in terms of the onerous administrative aspects required to consent patients and order WGS tests; 3) that the mainstreaming agenda, whilst considered important, encountered reluctance to become engaged from those who did not see it as a priority, or viewed it as being politically rather than clinically driven; and 4) that both the timelines and targets set for WGS were perceived by some of those delivering the service as overly-ambitious, creating tensions between those commissioning and those delivering the service.

Many of the challenges that we identified are not necessarily unique to the English NHS. For example, in the United States, common challenges have included improving clinicians’ knowledge and beliefs about genomic medicine, availability of genetic counsellors as well as medical and laboratory geneticists, and the computing infrastructure capabilities needed to analyze, store and share vast amounts of genomic data within and across healthcare systems (Sperber et al., 2017; Zebrowski et al., 2019; National Academies, 2023). In Australia, clinicians were found to be cautious about embracing the changes required for implementing genomic medicine. Specifically, not all clinicians felt comfortable about their knowledge and understanding of genomics, clinicians were hampered by a lack of time and funding, and there was a lack of interest amongst some clinicians, particularly older ones, for whom genomics was not considered a priority (Best et al., 2021b; Best et al., 2021c). Moreover, our findings echo many of the concerns raised previously by health professionals in the UK recruiting for the 100,000 Genomes Project. These include concern that WGS has not been demonstrated to be superior to exome sequencing for certain rare diseases, concerns around the time required to seek consent from patients, and concerns about whether adequate resources are in place to deliver a WGS service in a timely way (Sanderson et al., 2019). Regarding the superiority of WGS over exome sequencing, the issue is complex as WGS has the potential to improve diagnostic yield over exome sequencing through improved coverage of economic regions, ability to detect variants in non-coding regions and the mitochondrial genome, expansion variants, as well as more robust detection of some structural variants (Mattick et al., 2018). However, at the same time the full clinical potential of WGS is not being realized because analysis is in most cases restricted to a subset of genes on a virtual panel (rather than a gene agnostic WGS assay interrogation) to reduce off-target noise and incidental findings. However, this can result in missed diagnoses because of genetic heterogeneity (Wright et al., 2018).

Interviewees discussed several strategies and local adaptations that had been made to address the various challenges and bottlenecks they had encountered. For example, some sites had hired what are being referred to as “genomic associates” to support the consent process, enabling clinicians to focus their time on more specialist tasks. Genomic associates are an entirely new cadre of healthcare workers introduced without prior planning, to support the provision of WGS. As such, there is no formal career structure, training or qualification for this group of professionals, and no clear guidance on what the remit of their role should be, i.e., whether they should be tasked with discussing WGS and taking consent or only focusing on administrative aspects. Guidance in this area is therefore essential.

Some sites spoke of developing local training routes to upskill existing staff such as trainee clinical scientists, to support the demand for genomic testing. There were also examples of some GMSAs co-creating services with patients and the public, for example, through their participation on patient advisory boards, contributing to business plans and strategies as well as co-creating patient-friendly resources. There had been a concerted effort across all GLHs and GMSAs to improve clinician knowledge and buy-in of genomics and to upskill clinicians, although these were being delivered in different ways, including through education sessions, deploying genomic associates or genetic counsellors to train clinical teams, identifying a key person to act as a “genomics champion” within a mainstream departments who could impart knowledge and best practice, and the formation of regional MDTs to enhance collaboration between genetic and mainstream specialties. Interviewees reflected on improved collaboration and networking across the GLHs and GMSAs as well as the emergence of collaborative networks set up by NHS England and NHS Improvement to progress research and increase knowledge. Capacity-building, enhancing collaboration between genetic and mainstream specialties and co-creating services with patients and the public have all been identified as key to the successful implementation of genomics (Sperber et al., 2017; Long et al., 2019; Zimani et al., 2021) and are stated as priority actions by NHS England (NHS England, 2022b).

Outside of workforce shortages, not only within genomic services but across the NHS more generally, many of the barriers identified in this study are likely solvable through digital solutions, in particular those barriers identified at the test ordering stage as well as sample collection, analysis and reporting stages. These include the current reliance on paper-based forms, the lack of an end-to-end system enabling sample tracking, working across multiple sites, and adding results to patients’ electronic health records. Data and digital developments in terms of infrastructure and interoperability are a key enabler for the GMS and the difficulties in this area are due in part to the lack of standardization and hence variety of different informatics systems used across the NHS with varying digital maturity. In response to ongoing challenges in this area, NHSE have recently established a Genomics Data and Digital Board (established in February 2023) to coordinate the approach to supporting interoperability across NHS systems and drive efficiencies in the service (NHS England, 2022b).

Our findings need to be considered in light of the current state of the NHS which is already running at capacity and experiencing chronic understaffing, poor retention, insufficient funding and long waiting lists (British Medical Association, 2023). Moreover, the direct and indirect impacts of COVID-19 are still emerging but have undoubtedly exacerbated an already stretched healthcare system (GOV.UK, 2022; Nuffield Trust, 2022). It is therefore not surprising that the introduction of a new service, which requires but is generally hesitant to system-wide change, creates imminent challenges and may encounter mixed reactions from those tasked with delivering it. Yet one must also bear in mind that a great deal of progress has been made despite these challenges. Since its launch nearly 50,000 whole genomes have been sequenced, the vast majority (86%) in rare diseases with a diagnostic yield of around 33%, and over 150 clinical pathways are open for WGS (data given at presentation by Professor Dame Sue Hill at the PHG 25th Anniversary Conference, April 2023). Whilst many of the challenges identified through this work will undoubtedly be addressed over time, it will be vital to continue to build the evidence base to demonstrate the benefits of genomics to patients, professionals and the public more broadly. This includes not just the clinical benefits of genomic testing (Shickh et al., 2021), but also the psychological and social benefits (Robinson et al., 2019), which have been shown to be of greater importance to parents in some instances (Peter et al., 2022). Behavioral science research to capture experiences, decision-making, communication and outcomes for patients, families and health professionals is crucial in that regard in order to provide the evidence to inform policy and practice decisions about service delivery.

Our study was able to capture the experiences of those designing and delivering the GMS at unique point in time during the early years of the service. Given the fast-moving pace of change occurring, in particular the adaptations that are being made at a local level (for example, employing genomics associates to support the consenting process and administrative aspects) some of the identified barriers may no longer be (as) relevant. We tried to ensure comprehensive inclusion criteria by including participants from every regional GLH/GMSA, however we did not include genetic professionals in non-leadership roles who may have different views or experiences, nor did we include any non-genetic specialists in this study (i.e., “mainstream clinicians”). Capturing the views of these groups will also be important as will assessing the experiences of patients, parents and families. During the time when we were conducting the analysis, the CFIR was updated and included revisions to existing domains and constructs as well as the addition, removal, or relocation of constructs (Damschroder et al., 2022). As we had already begun data analysis, we continued to use the original version of the CFIR, but the revised version may potentially provide a more nuanced analysis of the data.

Genomic medicine has clear benefits for patients and families. However, establishing the effectiveness of this new technology is not sufficient to guarantee its uptake in routine clinical care. To ensure the success of the service, buy-in across all stakeholder groups will be vital. Our study has highlighted the complexities and challenges of implementing genomic medicine at a national level and also identifies some of the local as well as national strategies being employed to address these challenges. The work provides valuable evidence to those involved in designing services here in England so that insights can lead to action. The findings will also be of relevance to those in other countries embarking on their own genomic medicine programs. The challenges involved in delivering a genomic medicine service are clear; all of us working within this sphere have a part to play in ensuring its success.

## Data Availability

The raw data supporting the conclusion of this article will be made available by the authors, without undue reservation.

## References

[B1] Barwell J. Snape K. Wedderburn S. (2019). The new genomic medicine service and implications for patients. Clin. Med. Lond. Engl. 19 (4), 273–277. 10.7861/clinmedicine.19-4-273 PMC6752257 31308102

[B2] Bauer M. S. Damschroder L. Hagedorn H. Smith J. Kilbourne A. M. (2015). An introduction to implementation science for the non-specialist. BMC Psychol. 3 (1), 32. 10.1186/s40359-015-0089-9 26376626 PMC4573926

[B3] Best S. Brown H. Lunke S. Patel C. Pinner J. Barnett C. P. (2021a). Learning from scaling up ultra-rapid genomic testing for critically ill children to a national level. NPJ genomic Med. 6 (1), 5. 10.1038/s41525-020-00168-3 PMC7843635 33510162

[B4] Best S. Brown H. Stark Z. Long J. C. Ng L. Braithwaite J. (2021b). Teamwork in clinical genomics: a dynamic sociotechnical healthcare setting. J. Eval. Clin. Pract. 27 (6), 1369–1380. 10.1111/jep.13573 33949753

[B5] Best S. Long J. C. Gaff C. Braithwaite J. Taylor N. (2021c). Organizational perspectives on implementing complex health interventions: clinical genomics in Australia. J. health Organ. Manag. ahead-of-print(ahead-of-print), 825–845. ahead-of-print. 10.1108/jhom-12-2020-0495 34283896

[B6] British Medical Association (2023). An NHS under pressure. Available at https://www.bma.org.uk/advice-and-support/nhs-delivery-and-workforce/pressures/an-nhs-under-pressure (Accessed June 1, 2023).

[B7] Damschroder L. J. Aron D. C. Keith R. E. Kirsh S. R. Alexander J. A. Lowery J. C. (2009). Fostering implementation of health services research findings into practice: a consolidated framework for advancing implementation science. Implement Sci. 4, 50. 10.1186/1748-5908-4-50 19664226 PMC2736161

[B8] Damschroder L. J. Reardon C. M. Widerquist M. A. O. Lowery J. (2022). The updated Consolidated Framework for Implementation Research based on user feedback. Implement. Sci. 17 (1), 75–16. 10.1186/s13012-022-01245-0 36309746 PMC9617234

[B9] Genomics England (2017). The 100,000 genomes project protocol. 3 ed London: Genomics England Ltd.

[B10] Genomics England (2020). Genomics England and Illumina partner to deliver whole genome sequencing for england’s NHS genomic medicine service. Available at: https://www.genomicsengland.co.uk/news/genomics-england-and-illumina-sequence-whole-genomes-for-nhs-gms (Accessed July 13, 2023).

[B11] GOV.UK (2022). in Direct and indirect health impacts of COVID-19 in England: emergine Omicrom impacts. Care, D.O.H.A.S (Ed.).

[B12] GOV.UK (2023). in England rare disease action plan 2023: main report. Care, D.O.H.A.S (Ed.).

[B13] Kamps R. Brandão R. D. Bosch B. J. Paulussen A. D. Xanthoulea S. Blok M. J. (2017). Next-generation sequencing in oncology: genetic diagnosis, risk prediction and cancer classification. Int. J. Mol. Sci. 18 (2), 308. 10.3390/ijms18020308 28146134 PMC5343844

[B14] Long J. C. Pomare C. Best S. Boughtwood T. North K. Ellis L. A. (2019). Building a learning community of Australian clinical genomics: a social network study of the Australian Genomic Health Alliance. BMC Med. 17 (1), 44. 10.1186/s12916-019-1274-0 30791916 PMC6385428

[B15] Mattick J. S. Dinger M. Schonrock N. Cowley M. (2018). Whole genome sequencing provides better diagnostic yield and future value than whole exome sequencing. Med. J. Aust. 209 (5), 197–199. 10.5694/mja17.01176 29621958

[B16] National Academies (2023). Realizing the potential of genomics across the continuum of precision healthcare. 37011115

[B17] NHS England (2022a). Accelerated access collaborative board meeting papers: delivering innovation through the NHS genomic medicine service.

[B18] NHS England (2022b). Accelerating genomic medicine in the NHS: a strategy for embedding genomics in the NHS over the next 5 years.

[B19] Nuffield Trust (2022). How much is Covid-19 to blame for growing NHS waiting times? Available at: https://www.nuffieldtrust.org.uk/resource/how-much-is-covid-19-to-blame-for-growing-nhs-waiting-times (Accessed June 1, 2023).

[B20] Pearce C. Goettke E. Hallowell N. McCormack P. Flinter F. McKevitt C. (2019). Delivering genomic medicine in the United Kingdom National Health Service: a systematic review and narrative synthesis. Genet. Med. official J. Am. Coll. Med. Genet. 21 (12), 2667–2675. 10.1038/s41436-019-0579-x 31186523

[B21] Peter M. Hammond J. Sanderson S. C. Gurasashvili J. Hunter A. Searle B. (2022). Participant experiences of genome sequencing for rare diseases in the 100,000 Genomes Project: a mixed methods study. Eur. J. Hum. Genet. EJHG 30 (5), 604–610. 10.1038/s41431-022-01065-2 35264738 PMC9091267

[B22] Raspa M. Moultrie R. Toth D. Haque S. N. (2021). Barriers and facilitators to genetic service delivery models: scoping review. Interact. J. Med. Res. 10 (1), e23523. 10.2196/23523 33629958 PMC7952239

[B23] Roberts K. Dowell A. Nie J. B. (2019). Attempting rigour and replicability in thematic analysis of qualitative research data; a case study of codebook development. BMC Med. Res. Methodol. 19 (1), 66. 10.1186/s12874-019-0707-y 30922220 PMC6437927

[B24] Robinson J. O. Wynn J. Biesecker B. Biesecker L. G. Bernhardt B. Brothers K. B. (2019). Psychological outcomes related to exome and genome sequencing result disclosure: a meta-analysis of seven Clinical Sequencing Exploratory Research (CSER) Consortium studies. Genet. Med. official J. Am. Coll. Med. Genet. 21 (12), 2781–2790. 10.1038/s41436-019-0565-3 PMC7260995 31189963

[B25] Roden D. M. McLeod H. L. Relling M. V. Williams M. S. Mensah G. A. Peterson J. F. (2019). Pharmacogenomics. Lancet (London, Engl. 394 (10197), 521–532. 10.1016/s0140-6736(19)31276-0 PMC6707519 31395440

[B26] Sanderson S. C. Hill M. Patch C. Searle B. Lewis C. Chitty L. S. (2019). Delivering genome sequencing in clinical practice: an interview study with healthcare professionals involved in the 100 000 Genomes Project. BMJ open 9 (11), e029699. 10.1136/bmjopen-2019-029699 PMC6858183 31685495

[B27] Shickh S. Mighton C. Uleryk E. Pechlivanoglou P. Bombard Y. (2021). The clinical utility of exome and genome sequencing across clinical indications: a systematic review. Hum. Genet. 140 (10), 1403–1416. 10.1007/s00439-021-02331-x 34368901

[B28] Smedley D. Smith K. R. Martin A. Thomas E. A. McDonagh E. M. Cipriani V. (2021). 100,000 genomes pilot on rare-disease diagnosis in health care - preliminary report. N. Engl. J. Med. 385 (20), 1868–1880. 10.1056/NEJMoa2035790 34758253 PMC7613219

[B29] Sperber N. R. Carpenter J. S. Cavallari L. H. Cooper-DeHoff R. M. Denny J. C. Orlando L. A. (2017). Challenges and strategies for implementing genomic services in diverse settings: experiences from the Implementing GeNomics in pracTicE (IGNITE) network. BMC Med. genomics 10 (1), 35. 10.1186/s12920-017-0273-2 28532511 PMC5441047

[B30] Stark Z. Dolman L. Manolio T. A. Ozenberger B. Hill S. L. Caulfied M. J. (2019). Integrating genomics into healthcare: a global responsibility. Am. J. Hum. Genet. 104 (1), 13–20. 10.1016/j.ajhg.2018.11.014 30609404 PMC6323624

[B31] Turnbull C. Scott R. H. Thomas E. Jones L. Murugaesu N. Pretty F. B. (2018). The 100 000 Genomes Project: bringing whole genome sequencing to the NHS. Bmj 361, k1687. 10.1136/bmj.k1687 29691228

[B32] Walton N. A. Hafen B. Graceffo S. Sutherland N. Emmerson M. Palmquist R. (2022). The development of an infrastructure to facilitate the use of whole genome sequencing for population health. J. personalized Med. 12 (11), 1867. 10.3390/jpm12111867 PMC9693138 36579594

[B33] Weaver K. (2018). “Pragmatic paradigm,” in The SAGE encyclopedia of educational research, measurement, and evaluation. Editor Frey B. B. (Thousand Oaks: SAGE Publications, Inc).

[B34] Wright C. F. FitzPatrick D. R. Firth H. V. (2018). Paediatric genomics: diagnosing rare disease in children. Nat. Rev. Genet. 19 (5), 253–268. 10.1038/nrg.2017.116 29398702

[B35] Zebrowski A. M. Ellis D. E. Barg F. K. Sperber N. R. Bernhardt B. A. Denny J. C. (2019). Qualitative study of system-level factors related to genomic implementation. Genet. Med. official J. Am. Coll. Med. Genet. 21 (7), 1534–1540. 10.1038/s41436-018-0378-9 PMC6533158 30467402

[B36] Zimani A. N. Peterlin B. Kovanda A. (2021). Increasing genomic literacy through national genomic projects. Front. Genet. 12, 693253. 10.3389/fgene.2021.693253 34456970 PMC8387713

