# Supplementary material for: “A very big challenge”: a qualitative study to explore the early barriers and enablers to implementing a national genomic medicine service in England
Source: Front Genet. 2024 Jan 4;14:1282034. doi: 10.3389/fgene.2023.1282034 (PMC10794539; doi:10.3389/fgene.2023.1282034)
Supplement: Supplementary file 1 [file DataSheet2.docx]

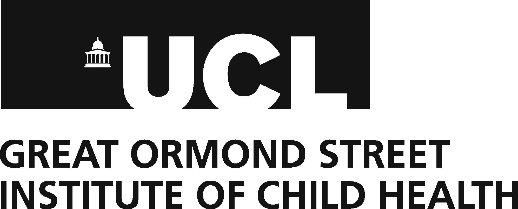


**Study title: A patient-centred evaluation of the new NHS Genomic Medicine Service**

**Implementation interviews with implementers and designers (Study 1)**

- *Introduce self*
- *Explain the purpose of the interview*

Your interview will help us to better understand the resources and activities that have been put in place to implement the new NHS genomic medicine service and to understand the intended outputs, outcomes and impact of the GMS.

- *Explain how the data will be used*

This interview will be recorded so that we have an accurate record of your thoughts.

***FOR TEAMS INTERVIEWS:*** *If you would prefer for just audio rather than video to be recorded then feel free to turn the video off.* Please be assured that the recording and your transcript will be kept confidential. Once your interview has been transcribed, it will be given an identifier and your name will not appear anywhere on the transcript. However, certain phrases or sentences may be used in academic publications or conferences.

Before we start, do you have any questions for me? *[Answer any questions]*

I am now going to turn on the audio/video recorder and go through the consent form so that I can record your consent verbally. I will go through each item one by one and please let me know if you are happy with each item by just saying yes or no.

- *Turn recorder on. Read each item aloud*

**Participant characteristics**

Before we start the main interview I am just going to collect some information about you. Please can you tell me:

1. Age
2. Organisation
3. Professional background
4. Current role
5. Years in role

**Introduction**

1. Could you start by giving me a description of your current role?
2. What role have you played in designing and implementing the Genomic Medicine Service (GMS)?

**Intervention characteristics / programme theory**

1. What is the purpose (overall aims) of the GMS in the context of rare disease diagnosis? (IC - intervention source)
   1. What was the rationale for the development of the GMS? (IC - intervention source)
   2. How does it improve on or build on what was available before? (IC - relative advantage)
2. What does the GMS seek to achieve?
   1. What do you feel are the main short term goals of the new GMS? (e.g. early detection and treatment, ending diagnostic odyssey, increased access to genomic testing, participate in research)
   2. What are the longer term goals? (e.g. build workforce literate in genetics, build national genomic knowledge base to inform academic/industry research)

**Main activities/components of the GMS**

1. Can you describe the system-level structures that have been set up as part of the GMS? (probe: Genomic Laboratory Hubs (GLHs); Genomic Medicine Centers (GMCs); Genomic Medicine Service Alliances (GMSAs); National genomic test directory; Genomics Clinical Reference Groups (CRGs) National Genomic Information System (NGIS))

**Preparing the workforce**

1. How have you prepared the workforce for adopting genetic/genomic testing in their clinical practice? (IS - readiness for implementation)
   1. What education and training has been put in place for staff? (IS - access to knowledge/information)
   2. What services or support is available for helping staff manage the practical/system aspects e.g. patient registration, sample processing etc [IC - Design quality and packaging]
   3. Are there sufficient resources in terms of clinical scientists, funding etc to implement genomic medicine? (IS - readiness for implementation - available resources)
2. How significantly different is the process of offering genomic testing going to be compared to how it was offered before? Will it be easier or more complex to order tests?( IC - complexity)

**Mainstreaming**

1. How have you tried to create behaviour/attitude change so that non-genetic specialists invest in the GMS? *(*IS - implementation climate ALSO Process - engaging key stakeholders)
   1. What steps have been taken to encourage non-genetic specialists to commit to using the GMS?(prompt: financial, other incentives) (OS - external policies and incentives)
   2. What have been the most successful strategies to enhance participation/engagement amongst non-genetic specialists? (IS - implementation climate ALSO Process - engaging key stakeholders))
   3. What kind of supporting evidence or proof is needed about the effectiveness of genomic medicine to get staff on board? (IC - evidence strength and quality)
2. What else do you feel could/should be done (if anything) to facilitate the mainstreaming of genomic medicine? (OS - external policies and incentives)

**Serving patients**

1. How well do you think the GMS will serve the needs of patients with rare undiagnosed conditions? (OS - patients’ needs and resources)
   1. What resources have been developed to support patients and families making decisions about genomic testing? (OS - patients’ needs and resources)
   2. Is there a national approach to address equity of access, particularly for harder to reach groups? (OS - patients’ needs and resources)

**Readiness**

1. How ready is the NHS for implementation of the GMS? (IS - readiness for implementation)
2. What kind of infrastructure changes are needed on a local level to accommodate the NHS GMS? (IS - changes)
3. Do you anticipate any differences in how GMSs will deliver the service? (IC - adaptability)

**Engagement**

1. How are those designing and implementing the GMS supporting or engaging with the staff who will be delivering it? (Process - readiness for implementation – leadership engagement)
2. Who are the key individuals at individual organization’s that you need to get on board with this new Service? (Process – engaging – opinion leaders/formally appointed implementation leaders]
3. Are there other people who are likely to champion (go above and beyond what might be expected) the GMS? (Process – engaging – Champions)

**Barriers and challenges**

1. Are there any particular barriers or challenges that you are currently facing in terms of implementing the GMS? Prompts: educating colleagues, coordinating care pathways, negotiating eligibility, returning results, costs and resource limitations (Process – executing)
   1. How are you trying to address those barriers or challenges? (Process – executing)
2. Do you foresee any specific ‘local factors’ at individual sites that might create barriers or challenges to them engaging in genomic medicine? (Process – executing)
3. What has been the impact of Covid on the roll-out of the GMS? (Process – executing)
4. Are there any particular barriers or challenges you anticipate in the future? (Process – planning)

**Evaluation**

1. How will you assess progress towards implementation or intervention goals NHS GMS? (Process – Reflecting and Evaluating)
2. What kind of information do you plan to collect as you implement the NHS GMS?  (Process – Reflecting and Evaluating)

23. Is there anything else you would like to share with me that I have not asked you?

*Topic guide informed through:*

- *CFIR: https://cfirguide.org/evaluation-design/qualitative-data/*
- *Taylor et al. 2019; A transformative translational change programme to introduce genomics into healthcare: a complexity and implementation science study protocol; BMJ Open*
- *Barwell et al. 2019; The new genomic medicine service and implications for patients; Clin Med*
