# Supplementary material for: “A very big challenge”: a qualitative study to explore the early barriers and enablers to implementing a national genomic medicine service in England
Source: Front Genet. 2024 Jan 4;14:1282034. doi: 10.3389/fgene.2023.1282034 (PMC10794539; doi:10.3389/fgene.2023.1282034)
Supplement: Supplementary file 2 [file DataSheet1.docx]

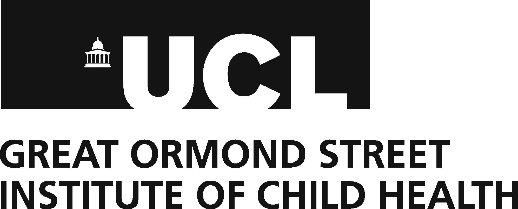


**Study title: A patient-centred evaluation of the new NHS Genomic Medicine Service**

**Implementation interviews with implementers and designers (Study 1)**

- *Introduce self*
- *Explain the purpose of the interview*

Your interview will help us to better understand the resources and activities that have been put in place to implement the new NHS genomic medicine service and to understand the intended outputs, outcomes and impact of the GMS.

- *Explain how the data will be used*

This interview will be recorded so that we have an accurate record of your thoughts.

***FOR TEAMS INTERVIEWS:*** *If you would prefer for just audio rather than video to be recorded then feel free to turn the video off.* Please be assured that the recording and your transcript will be kept confidential. Once your interview has been transcribed, it will be given an identifier and your name will not appear anywhere on the transcript. However, certain phrases or sentences may be used in academic publications or conferences.

Before we start, do you have any questions for me? *[Answer any questions]*

I am now going to turn on the audio/video recorder and go through the consent form so that I can record your consent verbally. I will go through each item one by one and please let me know if you are happy with each item by just saying yes or no.

- *Turn recorder on. Read each item aloud*

**Participant characteristics**

Before we start the main interview I am just going to collect some information about you. Please can you tell me:

1. Age
2. Organisation
3. Professional background
4. Current role
5. Years in role

**Introduction**

1. Could you start by giving me a description of your role in the GMSA/GLH

**Intervention characteristics / programme theory**

1. What is the purpose (overall aims) of the GMS in the context of rare disease diagnosis? (IC - intervention source)
   1. What was the rationale for the development of the GMS? (IC - intervention source)
   2. How does it improve on or build on what was available before? (IC - relative advantage)
2. What does the GMS seek to achieve?
   1. What do you feel are the main short term goals of the new GMS? (e.g. early detection and treatment, ending diagnostic odyssey, increased access to genomic testing, participate in research)
   2. What are the longer term goals? (e.g. build workforce literate in genetics, build national genomic knowledge base to inform academic/industry research)
3. What needs to happen to achieve these aims?

**Readiness**

1. Can you describe what has been done in your region to prepare for the GMS?
   1. Infrastructure changes (IS - changes)
   2. Education and training of the workforce
   3. To address equity of access
   4. Preparing and supporting patients

**Barriers and challenges**

1. What have been the main barriers or challenges that you have faced in terms of setting up the GMS? Prompts:
   1. reconfiguration
   2. costs and resource limitations (Process – executing)
   3. Space and equipment
2. What are the current barriers or challenges you are experiencing?
   1. Technical infrastructure – for consent, sample collection and tracking, interpretation of variant pipelines etc
   2. When requesting managerial support given acute and social care strains on hospital finances?
3. How are you trying to address those barriers or challenges? (Process – executing)
4. What has been the impact of Covid on the roll-out of the GMS? (Process – executing)

**Facilitators**

1. What has helped facilitate the implementation of the GMS (Process – executing)?
   1. Did the support of Clinical Research Network help when this became available?
2. Has anything been smoother than expected? (Process – executing)

**WGS**

1. What has been your experience with the roll-out of WGS for rare disease diagnosis?
   1. What have you done in terms of supporting clinicians who are/will be offering WGS?
   2. Have there been any challenges with offering WGS as a clinical service?
   3. Have you had to ‘ration’ the service in any way?
   4. Are we offering WGS for the right clinical indications?
   5. Consenting patients to participating in research?
2. What are your impressions of how well mainstreaming of WGS is going?
   1. Are they consenting patients?
   2. Which clinical specialties are referring patients for WGS?

**Workforce preparation**

1. How prepared are the workforce in your region for adopting genomic medicine in their clinical practice? (IS - readiness for implementation, implementation climate)
   1. What education and training has been put in place for staff? (IS - access to knowledge/information)
2. How are genetics professionals supporting their non-genetic colleagues as the service is established?
3. What else do you feel could/should be done (if anything) to facilitate the mainstreaming of genomic medicine? (OS - external policies and incentives)

**Engagement**

1. How much interaction has there been between those at a national level designing the GMS and those at a regional level delivering it? (Process - readiness for implementation – leadership engagement
2. What has been your contribution to the national strategy, if any? Are you involved in those discussions?

**Other**

1. Is there anything else you would like to share with me that I have not asked you?

*Topic guide informed through:*

- *CFIR: https://cfirguide.org/evaluation-design/qualitative-data/*
- *Taylor et al. 2019; A transformative translational change programme to introduce genomics into healthcare: a complexity and implementation science study protocol; BMJ Open*
- *Barwell et al. 2019; The new genomic medicine service and implications for patients; Clin Med*
